# Vibrations and energy distribution in inhomogeneous rods with elastic and viscous boundary conditions

**DOI:** 10.1038/s41598-024-52860-4

**Published:** 2024-02-03

**Authors:** János Lelkes, Bendegúz Dezső Bak, Tamás Kalmár-Nagy

**Affiliations:** Department of Fluid Mechanics, Faculty of Mechanical Engineering, Budapest University of Technology and Economics, Műegyetem rkp. 3., Budapest, 1111 Hungary

**Keywords:** Mechanical engineering, Applied mathematics

## Abstract

Functionally graded materials have broad engineering applications including mechanical engineering, electronics, chemistry, and biomedical engineering. One notable advantage of such materials is that their stiffness distribution can be optimized to avoid stress concentration. A novel approach for solving the equations describing the longitudinal vibration of functionally graded rods with viscous and elastic boundary conditions is proposed. The characteristic equation of the system is derived for the solution of the undamped case for the constant stiffness rod. Then, a homotopy method is applied to compute the eigenvalues and mode shapes of graded rods for viscoelastic boundary conditions. The changes of the eigenvalues and mode shapes as function of the damping parameters are investigated. The optimal damping of the system is computed. It is shown that the qualitative behavior depends on the relation between the actual damping and the optimal damping of the system. The energy density distribution of graded rods is also discussed. An energy measure, the mean scaled energy density distribution is introduced to characterize the energy distribution along the rod in the asymptotic time limit. The significance of such a measure is that it reveals how the energy tends to distribute along the rod. It is shown that the energy distribution can be manipulated by changing the damping parameters. Qualitative changes depending on the relation between the actual damping and the optimal damping are highlighted.

## Introduction

Many natural and engineering processes involve vibrations and the associated energy transfer. An oft-cited engineering application of the study of vibrations and energy transfer is vibration reduction^1–3^. Simple examples of tools for passive vibration reduction are tuned mass-damper systems^4^ . Many studies investigate the problem of optimal damping, i.e., what value should the damping have to achieve the fastest decay of energy. Nakić^5^ summarizes the different approaches on finding the optimal damping. In this field, a frequently arising problem is the vibration of cantilevers, consoles, and poles, i.e., any structure that resembles a rod (having a significant length relative to its thickness) with considerable mass.

There are two approaches to describe the behavior of such rods: the discrete and continuum models. The discrete approach is the chain oscillator model. In the chain oscillator, the continuum is replaced by a finite number of masses that are connected through springs and dampers. With this approach, the vibration can be described by means of a system of ordinary differential equations^6^. Many interesting applications arose even from this simpler approach; problems related to mass-spring chains on a line (the 1D lattice) have been extensively studied in the past decades^7–12^ .

Rods can be described by various continuum models as well^13–19^ . Recently, complex rod models can include nonlinearity and/or graded material. For instance, Santo et al.^20^ investigated the harmonic response of vibrating homogeneous rods with nonlinear elastic boundary conditions. In this study, we investigate the mode shapes as well as the energy distribution of functionally graded (position-dependent stiffness) elastic rods with elastic and viscous boundary conditions. Continuous changes in the properties of the functionally graded materials have engineering relevance, since this material behavior yields a lot of engineering benefits, such as avoiding the occurrence of large shear stresses^21^ . For example, Shi et al.^22^ investigated carbon-nanotube-reinforced composite beams where different nanotube distributions were assumed including functionally graded distributions through the thickness of the beams. Other base models with functionally graded material distribution are also considered in recent literature, such as the work of Zhang and Liu^23^ who investigated moving rectangular plates and the work of Ghamkhar et al.^24^ that discusses a three-layered cylinder shaped shell in which the central layer consists of functionally graded material. The static and dynamic characteristics of functionally graded materials are favorable in many scientific and engineering fields, such as aerospace, automobile, electronics, optics, chemistry, biomedical engineering, nuclear engineering and mechanical engineering^25–29^.

The functionally graded elastic rod model in this work is formulated in a general way. This means that the model gives a general solution for the following cases: inhomogeneous and homogeneous rod with free-free, fixed-free, fixed-fixed, fixed-spring, fixed-damper, spring-spring or spring-damper boundary conditions. An initial-boundary value problem describes the inhomogeneous elastic rod model. We solve this viscous-elastic initial-boundary value problem for the longitudinal displacement of the rod. We calculate the eigenvalues and mode shapes of the system that consists of a rod attached to springs and dampers at one or both ends. Then we compute the mean energy distribution along the rod for different stiffness distributions and damping parameters to show the effects of these parameters.

The mathematical description of the vibration of continuum rods leads to a Sturm–Liouville problem^30,31^. The richness of the underlying dynamics is highlighted by the variety of papers dealing with the vibration of homogeneous or inhomogeneous rods with different types of boundary conditions (fixed, free, elastic or viscous). Previous works have involved the determination of the natural frequencies and mode shapes for different boundary conditions^32–35^.

One novelty of this study is that we propose a homotopy method to determine the eigenvalues for the general inhomogeneous and damped cases. The basic idea behind homotopy methods is that a known solution of a simple problem may continuously be “deformed” into a solution of a more complex problem. Such deformation is called a homotopy^36^. He^37,38^ was the first who applied homotopy method to solve boundary value problems. Chun and Sakthivel^39^ applied the homotopy perturbation method for solving the linear and nonlinear two-point boundary value problems and compared it with the Adomian Decomposition Method^40,41^ and the shooting method^42^. Since then a huge amount of literature discusses the possible applications of the homotopy method^43–49^.

We also note here that continuation based on the homotopy method can fail for some systems^50^. The steps of our homotopy method can be summed up as follows: we first solve the governing equations of the vibration for an undamped homogeneous rod for which the solution can be obtained analytically. Then the Sturm–Liouville problem of the homogeneous rod with elastic boundary conditions is homotopically changed into that of a functionally graded rod with elastic and viscous boundary conditions. This way we can compute the eigenvalues numerically for slightly inhomogeneous and weakly damped systems, then we can gradually proceed to strongly inhomogeneous and/or strongly damped systems. Eventually the eigenvalues for the desired parameter combinations are reached.

The significant advancement presented in this research lies in its detailed exploration of a more comprehensive scenario of longitudinally vibrating rods, which distinguishes it from prior studies in this domain. This research goes beyond the existing literature^22,32–35,51,52^ by examining rods with both elastic and viscous boundary conditions at their ends, coupled with the complexity of inhomogeneous stiffness throughout the rod’s length. This particular case represents a new avenue in the field that, as far as we are aware, has yet to be previously addressed. Additionally, this study makes a notable contribution by calculating the optimal damping parameters and mapping out the energy distribution for these rods in such a generalized setting. This extended analysis provides a deeper understanding of the dynamics of longitudinally vibrating rods, offering valuable insights for future research and practical applications.

This paper is structured as follows: in “The functionally graded elastic rod model” section we describe the investigated system that consists of a functionally graded elastic rod model that is attached to springs and dampers on both ends. We formulate the partial differential equation that describes the longitudinal vibrations of the system. In “Characteristic equation, mode shapes, particular solution” section it is shown how the formulated initial-boundary value problem can be solved and how the eigenvalues and mode shapes of the system are obtained. The difficulty in determining the mode shapes stems from the transcendental nature of the characteristic Eq. (10), which in general can only be solved numerically. In “The constant stiffness rod and graded rods” section we show the solution for the simple case of the constant stiffness rod ($k(x)\equiv 1$). We also show the first few eigenvalues of the system calculated by the novel homotopy approach as the function of the damping coefficients for graded rods. In “Optimal damping” section we briefly discuss the concept of optimal damping. Then, in “Energy and its distribution in the rod” section we define the energy measures that describe the energy distribution in the vibrating rod, e.g., the energy density of the rod. In “Energy density distribution of the damped system with varying stiffness” section we will use $\lambda _{\pm 1} =\alpha _{1}\pm i\omega _{1}$ (the rightmost eigenvalue) and the corresponding mode shape that are associated with the slowest decaying vibration component to compute a time-independent energy measure of the system. This energy measure characterizes the asymptotic behavior of the system, essentially showing us how the energy tends to distribute along the rod during free vibration. It is shown how the energy is distributed in different graded rods and it is demonstrated that the energy distribution in the rods can be manipulated by tuning the damping coefficients at the ends of the rod. This finding has potential applications in channeling vibrational energy. Further potential physical applications include elastic wave propagation and localization in band gap materials^53^ and utilizing multi-mode vibration absorption capability of metamaterial beams^54^. Finally, conclusions are drawn in “Conclusions” section.

A few additional subscripts are used in the paper that are fully descriptive, hence, they are not listed in this table.

## The functionally graded elastic rod model

**Figure 1:**
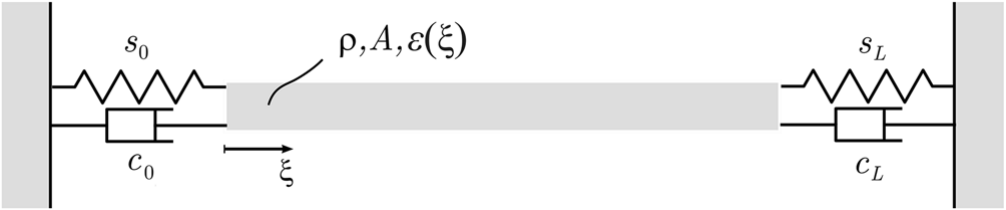
The continuous, functionally graded rod model with viscoelastic boundary conditions.

The longitudinal vibrations of an inhomogeneous, functionally graded rod, that is depicted in Fig. 1, are considered in the axial direction. The modulus of elasticity $\varepsilon (\xi )$ is a function of the axial coordinate $\xi$. The density $\rho$ and cross section *A* are constant along the rod. The left end of the rod at $\xi =0$ is connected to a spring of stiffness $s_{0}$ and a linear damper with damping parameter $c_{0}$. At the right end $\xi =L$, the rod is connected to a spring of stiffness $s_{L}$ and a linear damper with damping parameter $c_{L}$.

The governing differential equation with the above described boundary conditions is (for a detailed derivation see^51^)

1 $$\begin{aligned} \begin{aligned} \dfrac{\partial }{\partial \xi }\left[ \varepsilon (\xi )A\dfrac{\partial \phi (\xi ,\tau )}{\partial \xi }\right]&=\rho A\dfrac{\partial ^{2}\phi (\xi ,\tau )}{\partial \tau ^{2}},\\ \varepsilon _{0}A\left. \frac{\partial \phi (\xi ,\tau )}{\partial \xi }\right| _{\xi =0}&=s_{0}\phi (0,\tau )+c_{0}\left. \frac{\partial \phi (\xi ,\tau )}{\partial \tau }\right| _{\xi =0},\\ \varepsilon _{L}A\left. \frac{\partial \phi (\xi ,\tau )}{\partial \xi }\right| _{\xi =L}&=-s_{L}\phi (L,\tau )-c_{L}\left. \frac{\partial \phi (\xi ,\tau )}{\partial \tau }\right| _{\xi =L}. \end{aligned} \end{aligned}$$

where $\phi (\xi ,\tau )$ is the displacement in longitudinal direction, $\tau$ is the time, $\varepsilon _{0}=\varepsilon (0), \varepsilon _{L}=\varepsilon (L)$. The variation of the Young’s modulus is defined as $\varepsilon (\xi )=\varepsilon _{0}k(\xi )$, where $k(0)=1$.

The discussion of initial conditions is deferred to “Initial conditions and the particular solution” section.

To reduce the parameters of the system we introduce the nondimensional coordinate *x*, time *t* and displacement *u*(*x*, *t*) as $x=\frac{\xi }{L},\, t=\frac{\tau }{\Theta },\, u=\frac{\phi }{L}$, where *L* is the length of the rod and $\Theta$ is the timescale of the system. After nondimensionalization of Eq. (1) and setting $\Theta =L\sqrt{\frac{\rho }{\varepsilon _{0}}}$, we get

2 $$\begin{aligned} \begin{aligned} \dfrac{\partial }{\partial x}\left[ k(x)\dfrac{\partial u(x,t)}{\partial x}\right]&=\dfrac{\partial ^{2}u(x,t)}{\partial t^{2}},\\ \left. \frac{\partial u(x,t)}{\partial x}\right| _{x=0}&=\frac{s_{0} L}{\varepsilon _{0}A}u(0,t)+\frac{c_{0}}{\varepsilon _{0}A}\sqrt{\frac{\varepsilon _{0}}{\rho }}\left. \frac{\partial u(x,t)}{\partial t}\right| _{x=0},\\ \left. \frac{\partial u(x,t)}{\partial x}\right| _{x=1}&=-\frac{s_{L} L}{\varepsilon _{L}A}u(1,t)-\frac{c_{L}}{\varepsilon _{L}A}\sqrt{\frac{\varepsilon _{0}}{\rho }}\left. \frac{\partial u(x,t)}{\partial t}\right| _{x=1}. \end{aligned} \end{aligned}$$

For the sake of simplicity we set the dimensionless groups of the spring boundary conditions equal to 1, i.e., $\frac{s_{0}L}{\varepsilon _{0}A}=1,\frac{s_{L} L}{\varepsilon _{L}A}=1$. These choices allow us to match the stiffnesses of the springs with the stiffness of the rod at its two endpoints $x=0$, $x=1$. Thus, the force is the same regardless of whether the spring or the connecting endpoint of the rod is displaced by the same amount. Otherwise, these two dimensionless groups could be adjusted between 0 (free end) and infinity (fixed end). These cases are not covered in this paper.

We introduce the dimensionless stiffnesses as $k_{0}=\frac{s_{0}L}{\varepsilon _{0} A}=1,\, k_{1}=\frac{s_{L}L}{\varepsilon _{0}A}=\frac{s_{L}}{s_{0}}$, and the dimensionless damping coefficients as $d_{0}=\frac{c_{0}}{\varepsilon _{0}A}\sqrt{\frac{\varepsilon _{0}}{\rho }},\, d_{1}=\frac{c_{L} }{\varepsilon _{L}A}\sqrt{\frac{\varepsilon _{0}}{\rho }}$. Substituting the dimensionless stiffnesses and damping coefficients into Eq. (2), the dimensionless boundary value problem becomes

3 $$\begin{aligned} \begin{aligned} \ddot{{u}}(x,t)&={k}(x){u}^{\prime \prime }(x,t)+{k}^{\prime }(x){u}^{\prime }(x,t),\\ u^{\prime }(0,t)&=u(0,t)+d_{0}\dot{u}(0,t),\\ u^{\prime }(1,t)&=-u(1,t)-d_{1}\dot{u}(1,t). \end{aligned} \end{aligned}$$

where the dot and the prime denote the derivative with respect to the dimensionless time and space, respectively.

## Characteristic equation, mode shapes, particular solution

First we will determine the *general* solution of the problem, and later we determine the *particular* solution for given initial conditions.

Let us note that system (3) is not self-adjoint due to the form of the boundary conditions (see also^34^). Even though separation of variables can in general be used for self-adjoint problems, there are some exceptions (including the wave equation with impedance boundary conditions^55^).

With this caveat, we develop a decoupled series of ordinary differential equations that represent the boundary value problem (3), i.e., we formulate the solution *u*(*x*, *t*) by separation of variables. Our approach is similar to the derivation described in^32^, but we assume the general solution of Eq. (3) to have the infinite sum of products form (akin to the eigenfunction expansion technique)

4 $$\begin{aligned} u(x,t)=\sum \limits _{j=-\infty }^{\infty }u(x,t,\lambda _{j}). \end{aligned}$$

Each term of the sum in Eq. (4) describes a *partial solution* $u(x,t,\lambda _{j})$ associated with the eigenvalue $\lambda _{j}$ as

5 $$\begin{aligned} u(x,t,\lambda _{j})=U(x,\lambda _{j})T(t,\lambda _{j}). \end{aligned}$$

The space-dependent functions $U(x,\lambda _{j})$ are the *complex mode shapes* associated with the complex eigenvalues $\lambda _{j}$. Equation (4) is substituted into Eq. (3) to yield

6 $$\begin{aligned} \begin{aligned} \frac{\ddot{T}(t,\lambda _{j})}{T(t,\lambda _{j})}&=\frac{U^{\prime \prime }(x,\lambda _{j})}{U(x,\lambda _{j})}k(x)+\frac{U^{\prime }(x,\lambda _{j})}{U(x,\lambda _{j})}k^{\prime }(x),\\ U^{\prime }(0,\lambda _{j})&=U(0,\lambda _{j})+d_{0}U(0,\lambda _{j})\frac{\dot{T}(t,\lambda _{j})}{T(t,\lambda _{j})},\\ U^{\prime }(1,\lambda _{j})&=-U(1,\lambda _{j})-d_{1}U(1,\lambda _{j})\frac{\dot{T}(t,\lambda _{j})}{T(t,\lambda _{j})}. \end{aligned} \end{aligned}$$

The solution for the temporal part has the form

7 $$\begin{aligned} T\left( t,\lambda _{j} \right) =T(0,\lambda _{j})e^{\lambda _{j} t}, \end{aligned}$$

and thus $\frac{\ddot{T}(t,\lambda _{j})}{T(t,\lambda _{j})}=\lambda _{j}^{2}$. Substituting this result into Eq. (6) and rearranging the equation yields the boundary value problem

8 $$\begin{aligned} \begin{aligned}{}&k(x)U^{\prime \prime }(x,\lambda _{j})+k^{\prime }(x)U^{\prime }(x,\lambda _{j})-\lambda _{j}^{2}U(x,\lambda _{j})=0,\\&U^{\prime }(0,\lambda _{j})=U(0,\lambda _{j})\left( 1+d_{0}\lambda _{j}\right) ,\\&U^{\prime }(1,\lambda _{j})=-U(1,\lambda _{j})\left( 1+d_{1}\lambda _{j}\right) . \end{aligned} \end{aligned}$$

The boundary value problem (8) is a Sturm–Liouville problem with separated, but eigenparameter-dependent boundary conditions^30^. We emphasized the $\lambda _{j}$-dependence of *U* by writing $U\left( x,\lambda _{j}\right)$. Equation (8) is a second-order linear equation, its general solution $U(x,\lambda _{j})$ can be written as

9 $$\begin{aligned} U(x,\lambda _{j})=D_{1}\Psi _{1}(x,\lambda _{j})+D_{2}\Psi _{2}(x,\lambda _{j}), \end{aligned}$$

where the complex functions $\Psi _{1},\Psi _{2}$ and the constants $D_{1}$, $D_{2}$ depend on the choice of $k\left( x\right)$. By substituting Eq. (9) into the boundary conditions of Eq. (8) a matrix equation is obtained for the unknown constants $D_{1}, D_{2}$. To get a non-trivial solution for $D_{1}$ and $D_{2}$, the determinant of the matrix has to vanish. Computing the determinant yields the characteristic equation of the system:

10 $$\begin{aligned} \begin{aligned} P_g(\lambda _{j},d_0,d_1)&=\left( (1+d_{0}\lambda _{j})\Psi _{1}(0,\lambda _{j})-\Psi _{1}^{\prime }(0,\lambda _{j} )\right) \left( (1+d_{1}\lambda _{j})\Psi _{2}(1,\lambda _{j})+\Psi _{2}^{\prime }(1,\lambda _{j})\right) \\&-\left( (1+d_{0}\lambda _{j})\Psi _{2}(0,\lambda _{j})-\Psi _{2}^{\prime }(0,\lambda _{j} )\right) \left( (1+d_{1}\lambda _{j})\Psi _{1}(1,\lambda _{j})+\Psi _{1}^{\prime }(1,\lambda _{j})\right) =0, \end{aligned} \end{aligned}$$

where $P_g(\lambda _{j},d_0,d_1)$ is the characteristic polynomial of a graded rod. The characteristic Eq. (10) is, in general, a transcendental equation with a countably infinite set of complex roots $\lambda _{j}=\alpha _{j}\pm {\textrm{i}}\omega _{j}$ (called characteristic roots or eigenvalues). We order the characteristic roots based on the magnitude of their real parts as $\alpha _{0}\ge \alpha _{\pm 1}\ge \alpha _{\pm 2}\ge ...$, with $\lambda _{-j}=\bar{\lambda }_{j}$ where the overbar symbol stands for complex conjugation.

The complex mode shapes corresponding to the complex conjugate roots are also complex conjugates, i.e., $U(x,\lambda _{-j})=U(x,\bar{\lambda }_{j})=\bar{U}(x,\lambda _{j})$. Furthermore, using Eq. (7) and $\lambda _{-j}=\bar{\lambda }_{j}$ we have $T(t,\lambda _{-j})=T(t,\bar{\lambda } _{j})=\bar{T}(t,\lambda _{j})$. Making use of these complex conjugate relations, and substituting Eq. (7) for $T(t,\lambda _{j})$ the solution (4) can be written as

11 $$\begin{aligned} u(x,t)=\sum \limits _{j=-\infty }^{\infty }U(x,\lambda _{j})T_{j}(0){\textrm{e}}^{\lambda _{j}t}=\sum \limits _{j=0}^{\infty }T_{j}(0)\left( U(x,\lambda _{j}){\textrm{e}}^{\lambda} _{j}+\bar{U}(x,\lambda _{j}){\textrm{e}}^{\bar{\lambda}}_{j}t\right) =\sum \limits _{j=0}^{\infty }T_{j}(0)\varphi (x,t,\lambda _{j}), \end{aligned}$$

where $${\varphi }(x,t,\lambda _{j})=U(x,\lambda _{j}){\textrm{e}}^{\lambda} _{j}t+\bar{U}(x,\lambda _{j}){\textrm{e}}^{\bar{\lambda }}_{j}t$$ and $$T_{j}(0)\equiv T(0,\lambda _{j})$$.

The term ${\varphi }(x,t,\lambda _{j})$ in Eq. (11) can be reformulated with Euler’s formula (using $\lambda _{j}=\alpha _{j}\pm {\textrm{i}}\omega _{j}$) as

12 $$\begin{aligned} \begin{aligned} \varphi (x,t,\lambda _{j})&=U(x,\lambda _{j}){\textrm{e}}^{\lambda} _{j}t+\bar{U}(x,\lambda _{j}){\textrm{e}}^{\bar{\lambda}}_{j}t=U(x,\lambda _{j}){\textrm{e}}^{(\alpha _{j}+{\textrm{i}}\omega _{j})t}+\bar{U}(x,\lambda _{j}){\textrm{e}}^{(\alpha _{j}-{\textrm{i}}\omega _{j})t} \\&=U(x,\lambda _{j}){\textrm{e}}^{\alpha _{j}}\left[ {\textrm{cos}}(\omega _{j}t)+{\textrm{i}}\,\textrm{sin}(\omega _{j}t) \right] +\bar{U}(x,\lambda _{j}){\textrm{e}}^{\alpha _{j}}\left[ {\textrm{cos}}(\omega _{j}t)-{\textrm{i}}\,\textrm{sin}(\omega _{j}t) \right] \\&={\textrm{e}}^{\alpha _{j}}\left[ \left( U(x,\lambda _{j})+\bar{U}(x,\lambda _{j})\right) {\textrm{cos}}(\omega _{j}t)+\left( U(x,\lambda _{j})-\bar{U}(x,\lambda _{j})\right) {\textrm{i}}\,\textrm{sin}(\omega _{j}t)\right] \\&={\textrm{e}}^{\alpha _{j}t}\left[ W(x,\lambda _{j})\textrm{cos}(\omega _{j}t)+Q(x,\lambda _{j})\textrm{sin}(\omega _{j}t)\right] ,\\ W(x,\lambda _{j})&=U(x,\lambda _{j}){+\bar{U}}(x,\lambda _{j})=2{\text {Re}}U(x,\lambda _{j}),\\ Q(x,\lambda _{j})&={\textrm{i}}(U(x,\lambda _{j})-\bar{U}(x,\lambda _{j}))=-2{\text {Im}}U(x,\lambda _{j}), \end{aligned} \end{aligned}$$

where $W(x,\lambda _{j})$ and $Q(x,\lambda _{j})$ are the *real mode shapes*.

### Initial conditions and the particular solution

To obtain the particular solution of the problem we also need the (nondimensional) initial conditions

13 $$\begin{aligned} u(x,0)=f(x),\quad \dot{u}(x,0)=g(x). \end{aligned}$$

The initial functions *f*(*x*) and *g*(*x*) have to satisfy the boundary conditions (see Eq. (3)), i.e.,

14 $$\begin{aligned} f^{\prime }(0)=f(0)+d_{0}g(0),\quad f^{\prime }(1)=-f(1)-d_{1}g(1). \end{aligned}$$

A partial solution $u(x,t,\lambda _{j})$ cannot in general satisfy the initial conditions for $f(x),\,g(x)$. To obtain *u*(*x*, *t*) for given initial conditions $f(x),\,g(x)$, the $T_{j}(0)$ coefficients must be determined. To do this, we can use the relationship between $W(x,\lambda _{j})$, $Q(x,\lambda _{j})$ and the initial conditions $f(x),\,g(x)$, that can be determined by substituting the initial conditions (13) into (11) to yield

15 $$\begin{aligned} \begin{aligned} u(x,0)&=\sum \limits _{j=0}^{\infty }T_{j}(0)W(x,\lambda _{j})=f\left( x\right) ,\\ \dot{u}(x,0)&=\sum \limits _{j=0}^{\infty }T_{j}(0)(\alpha _{j}W(x,\lambda _{j})+\omega _{j}Q(x,\lambda _{j}))=g(x). \end{aligned} \end{aligned}$$

This equation shows that the initial functions are combinations of the mode shapes, arbitrarily chosen functions cannot satisfy Eq. (15). This does not restrict practical applicability of the method as any prescribed initial condition can be approximated with a combination of mode shapes.

## The constant stiffness rod and graded rods

### Solving the characteristic equation for the constant stiffness rod

A particularly simple case of the problem is when the stiffness distribution along the rod is constant, i.e., $k(x)\equiv 1$. The boundary-value problem (8) is now simplified and yields

16 $$\begin{aligned} \begin{aligned}{}&U^{\prime \prime }\left( x\right) -\lambda _{j}^{2}U(x)=0,\\&U^{\prime }(0)=U(0)(1+d_{0}\lambda _{j}),\\&U^{\prime }(1)=-U(1)(1+d_{1}\,\lambda _{j}). \end{aligned} \end{aligned}$$

A simple substitution of $\lambda _{j}=0$ into Eq. (16) shows that 0 is not an eigenvalue. The general solution of Eq. (16) is

17 $$\begin{aligned} U(x,\lambda _{j})=D_{1}{\textrm{e}}^{\lambda _{j} x}+D_{2}{\textrm{e}}^{-\lambda _{j} x}, \end{aligned}$$

and the characteristic Eq. (10) becomes

18 $$\begin{aligned} P_c(\lambda _{j},d_0,d_1)=\left( (d_{0}d_{1}+1)\lambda ^{2}+(d_{0}+d_{1})\lambda _{j}+1\right) \sinh (\lambda _{j})+\left( (d_{0}+d_{1})\lambda _{j}^{2}+2\lambda _{j}\right) \cosh (\lambda _{j})=0. \end{aligned}$$

where $P_c(\lambda _{j},d_0,d_1)$ is the characteristic polynomial of the constant stiffness rod. Equation (18) is a transcendental equation with countably infinite complex roots.

We note that Eq. (18) can have one or two negative real roots if the damping of the system reaches its critical value. The relation for the number of real roots is

19 $$\begin{aligned} \begin{aligned}{}&\text {no real}\,\lambda _{j}\,\,\,\textrm{if}\,\,0\le d_{0}\le 1\,\textrm{and}\,0\le d_{1}\le 1,\,\,\\&\text {one real}\,\lambda _{j}\,\,\,\textrm{if}\,\,(0\le d_{0}\le 1\,\textrm{and}\,1<d_{1})\,\,\,\textrm{or}\,\,\,(1<d_{0}\,\textrm{and}\,0\le d_{1}\le 1),\\&\text {two real}\,\lambda _{j}`s\,\,\,\textrm{if}\,\,1<d_{0}\,\textrm{and} \,1<d_{1}. \end{aligned} \end{aligned}$$

In this paper, we are interested in the oscillatory motions and time-varying energy distributions. Thus the overdamped behavior will not be investigated.

We solve Eq. (18) for the $d_{0}=d_{1}=0$ case. We know that the eigenvalues are purely complex in this case, i.e., $\lambda _{\pm j}=\pm {\textrm{i}}\omega _{j}$ and the characteristic equation becomes

20 $$\begin{aligned} \tan (\omega _{j})=\frac{2\omega _{j}}{\omega _{j}^{2}-1}. \end{aligned}$$

Even though this equation is also transcendental, it is easy to solve numerically to yield a set of $\omega _{j}$’s. We then apply a homotopy continuation method to determine the characteristic roots, i.e., the solutions of Eq. (18) of the damped system similarly to^36^. The dampings $d_{0},d_{1}$ are increased in small steps and the characteristic roots of Eq. (18) are calculated by Newton’s method with the roots determined in the previous step as the starting point of the root finding.

**Figure 2:**
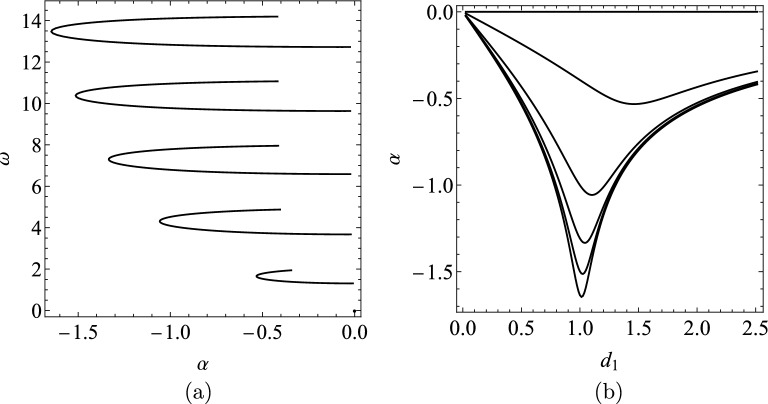
(**a**) Root locus plot for the characteristic Eq. (18) with constant rod stiffness $k(x)=1$ for $d_{1} \in \left[ 0,2.5\right]$, $d_{0}=0$. (**b**) Real part of the rightmost six complex eigenvalues as function of the damping $d_{1}$.

**Figure 3:**
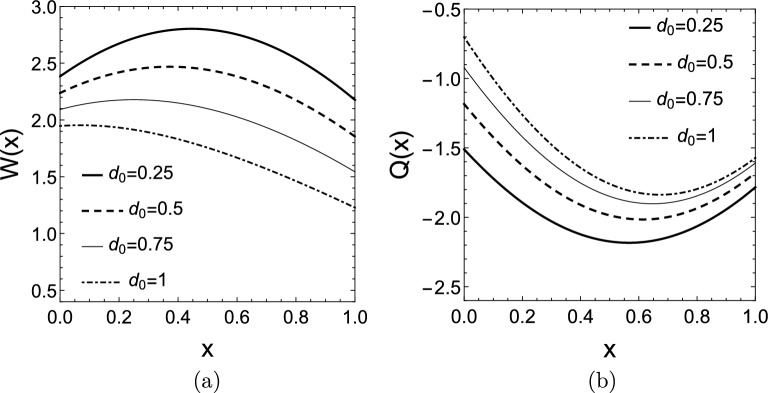
(**a**) First mode shape $W(x,\lambda _{1})$ and (**b**) $Q(x,\lambda _{1})$ with constant rod stiffness $k(x)=1$ for different damping values $d_{0}$, when $d_{1}=0$.

Figure 2 shows how the six rightmost complex characteristic roots ($\lambda _{j}$’s) change on the complex plane as the function of the damping $d_{1}\in \left[ 0,2.5\right]$, with $d_{0}=0$. The change of the real parts ($\alpha _{j}$’s) against the damping $d_{1}$ are also depicted here. In Fig. 3 the real mode shapes $W(x,\lambda _{1})$ and $Q(x,\lambda _{1})$ corresponding to $\lambda _{1}$ are depicted for $d_{0}\in \left\{ 0.25,0.5,0.75,1\right\}$ with $d_{1}=0$. These are calculated from the complex mode shapes $U(x,\lambda _{1})$ and $\bar{U}(x,\lambda _{1})$ using Eq. (12).

### Calculating the analytical solution for the constant stiffness rod

To determine the analytical solution *u*(*x*, *t*), the $\lambda$ eigenvalues, the $W(x,\lambda _{j})$ real mode shapes, and the $T_{j}(0)$ coefficients are needed. Since we already determined the eigenvalues and real mode shapes, we only need to determine the $T_{j}(0)$ coefficients.

To get the analytical solution *u*(*x*, *t*), the initial functions $f\left( x\right)$ and $g\left( x\right)$ need to be decomposed in the basis of the real mode shapes $W(x,\lambda _{j})$ as

21 $$\begin{aligned} f\left( x\right) \approx \sum \limits _{j=0}^{N}T_{j}(0)W(x,\lambda _{j}). \end{aligned}$$

Since this basis is non-orthogonal, we determine the coefficients $T_{j}(0)$ by minimizing the mean square error (MSE)

22 $$\begin{aligned} MSE=\int \limits _{0}^{1}\left( f(x)-\sum \limits _{j=0}^{N}T_{j}(0)W(x,\lambda _{j})\right) ^{2}{\textrm{d}}x. \end{aligned}$$

We discretize the initial displacement function and apply the least squares method to find the $T_{j}(0)$ coefficients of the real modes $W(x,\lambda _{j})$.

To illustrate this approximation of the initial displacement function we use two examples: (a) $f(x)=1+x-x^{2}$ and (b) $f(x)=1+x-x^{2} -10x^{3}+8x^{4}$. Using the first 12 modes decreases the error value to $10^{-6}$, while for 50 modes, the error is $5\cdot 10^{-7}$. Using more modes is not beneficial since we could not reduce the error to less than $10^{-7}$.

**Figure 4:**
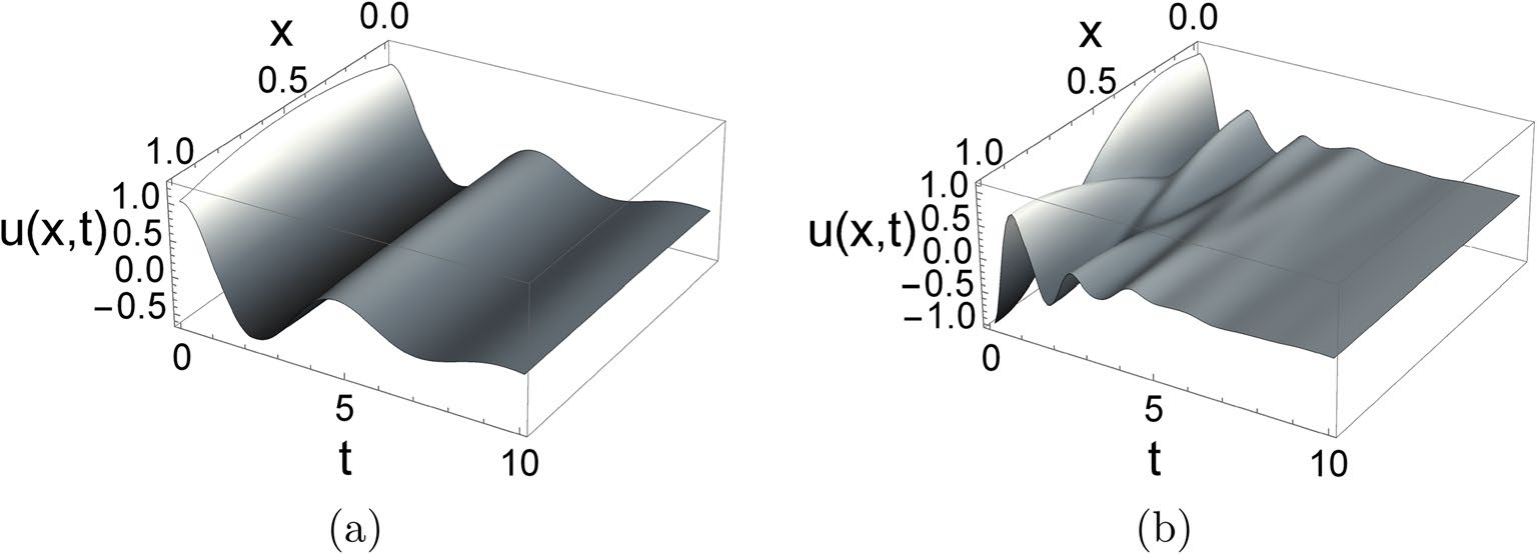
Solution *u*(*x*, *t*) corresponding to the 10 mode approximation of the initial displacement functions (**a**) $f(x)=1 + x - x^{2}$ and (**b**) $f(x)=1 + x - x^{2} - 10 x^{3} + 8 x^{4}$ for damping values $d_{0}=0.5$ and $d_{1}=0$.

The solutions *u*(*x*, *t*) corresponding to the 10-mode approximation of the initial displacement functions $f(x)=1+x-x^{2}$ and $f(x)=1+x-x^{2}-10x^{3}+8x^{4}$ are illustrated in Fig. 4 for $d_{0}=0.5$, $d_{1}=0$. In both cases, we observe that after the initial transient behavior, the first real mode becomes dominant. In further investigations, we will use the first dominant mode shape, which corresponds to the eigenvalue with the largest real part, i.e., its decay rate is the slowest.

### Rod with exponential stiffness distribution

An Example for the general solution of Eq. (8) for a particular stiffness distribution is provided in this section. We will present an exponential stiffness distribution as an example for a relatively extreme grading. We also carried out all of the following investigations for a linearly changing stiffness distribution as an example for a mild grading, and came to similar conclusions.

The step-by-step method to compute *u*(*x*, *t*) of the functionally graded rod with elastic and viscous boundary conditions for any *k*(*x*) is summarized as follows:

- From the boundary value problem (6) we construct the characteristic Eq. (10). The general solution (complex mode shapes) $U(x,\lambda _{j})$ is determined.
- The characteristic Eq. (20) for the constant stiffness rod is solved to obtain the first approximation of the eigenvalues.
- A homotopy method (described in the Supplementary Appendix) is used to determine the eigenvalues $\lambda _{j}$ corresponding to the arbitrary stiffness distribution *k*(*x*) and $d_{0},d_{1}$ damping values.
- The complex mode shapes $U\left( x,\lambda _{j}\right)$ are determined for *k*(*x*), $d_{0},d_{1}$.
- The real valued functions $W(x,\lambda _{j})$ and $Q(x,\lambda _{j})$ are determined using Eq. (12).
- The constant $T_{j}(0)$ are determined using Eq. (15) for given initial conditions $f(x),\, g(x)$.
- The real valued solution *u*(*x*, *t*) is provided based on Eqs. (11)–(12).

An example for a functionally graded elastic rod with closed-form mode shapes is when the stiffness distribution changes exponentially along the rod, i.e., $k(x)=e^{\mu x}$. The general solution of (8) can be computed with a symbolic computational tool (e.g., Wolfram Mathematica). For this exponentially varying stiffness it is

23 $$\begin{aligned} U(x,\lambda _{j})=D_{1}\frac{\lambda _{j}\sqrt{e^{-\mu x}}\left( K_{1}\left( \frac{2\sqrt{e^{-\mu x}}}{\mu }\lambda _{j}\right) \right) }{\mu }+D_{2} \frac{-\lambda _{j}\sqrt{e^{-\mu x}}\left( I_{1}\left( \frac{2\sqrt{e^{-\mu x}} }{\mu }\lambda _{j}\right) \right) }{\mu }, \end{aligned}$$

where $K_{1}$ and $I_{1}$ are the modified Bessel function of the second kind and the modified Bessel function of the first kind, respectively.

**Figure 5:**
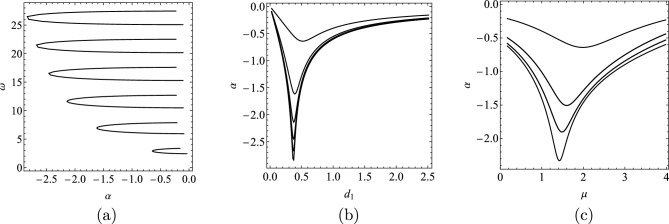
(**a**) Root locus plot for the characteristic Eq. (10) with exponentially changing rod stiffness $k(x)=e^{2x}$ for different damping values $d_{1}$, when $d_{0}=0$. (**b**) Real part of the rightmost six eigenvalues as function of the damping $d_{1}$. (**c**) Dependence of $\alpha$ on $\mu$ for exponentially varying stiffness with damping values $d_{0}=0,\,d_{1}=0.5$.

Figure 5a shows how the six rightmost characteristic roots change as the function of the damping $d_{1}$ for the exponential stiffness distribution $k(x)=e^{\mu x}$ with $\mu =2$. The other damping is set to zero, i.e. $d_{0}=0$. The dependence of the real parts on the damping coefficient $d_{1}$ are depicted separately in Fig. 5b. The dependence of the eigenvalues on the parameter $\mu$ was also investigated. Figure 5c shows the behavior of the real parts $\alpha _{j}$ as function of the parameter $\mu$ for fixed damping values $d_{0}=0,\,d_{1}=0.5$. The damping is added at the stiff end, the parameter $\mu$ has an optimal value where the decay rate of the vibration is the highest.

Due to the asymmetry of the stiffness distribution, setting the damping coefficients in the other way around yields different results. We also computed the case as the function of $d_{0}$ with $d_{1}=0$ this time. The eigenvalues on the complex plane and the real parts as function of the damping value showed similar trends, but the dependence on $\mu$ yielded a different result for the investigated damping value pair $d_{0}=1.5,\,d_{1}=0$. Even though we significantly extended the investigated parameter range for $\mu$ ($\mu \in [0,20]$ was investigated), we did not find any minimum, the real parts monotonously decrease as $\mu$ increases.

**Figure 6:**
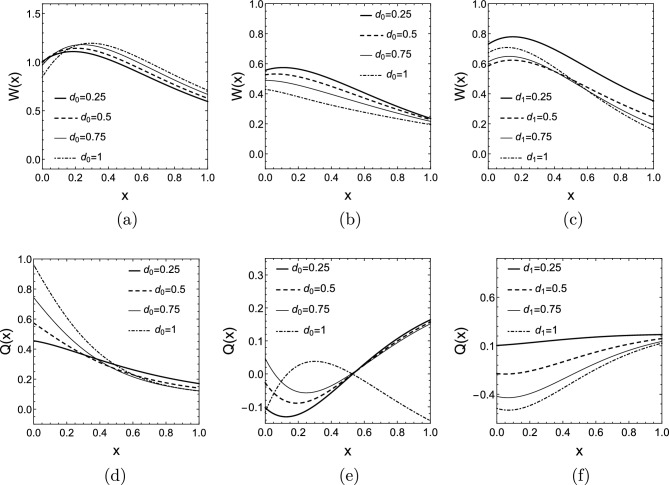
The first mode shape *W*(*x*) with exponential rod stiffness $k(x)=e^{2x}$ for (**a**) different damping values $d_{0}$, when $d_{1}=0$, (**b**) different damping values $d_{0}$, when $d_{1}=0.5$, and (**c**) different damping values $d_{1}$, when $d_{0}=0$. The first mode shape *Q*(*x*) with exponential rod stiffness $k(x)=e^{2x}$ for (**d**) different damping values $d_{0}$, when $d_{1}=0$, (**e**) different damping values $d_{0}$, when $d_{1}=0.5$, and (**f**) different damping values $d_{1}$, when $d_{0}=0$.

In Fig. 6 the real valued mode shapes $W(x,\lambda _{1})$ and $Q(x,\lambda _{1})$ are depicted for different damping combinations when $\mu =2$ is chosen, i.e., $k(x)=e^{2x}$. For simplicity, the initial functions *f*(*x*) and *g*(*x*) are specified by the dominant mode of the vibration, which are the first elements of the sums defined in (15) (the mode corresponding to the rightmost eigenvalue):

24 $$\begin{aligned} f(x)=T_1(0)W(x,\lambda _{1}),\quad g(x)=T_1(0)\left( \alpha _{1}W(x,\lambda _{1})+\omega _{1}Q(x,\lambda _{1})\right) . \end{aligned}$$

**Figure 7:**
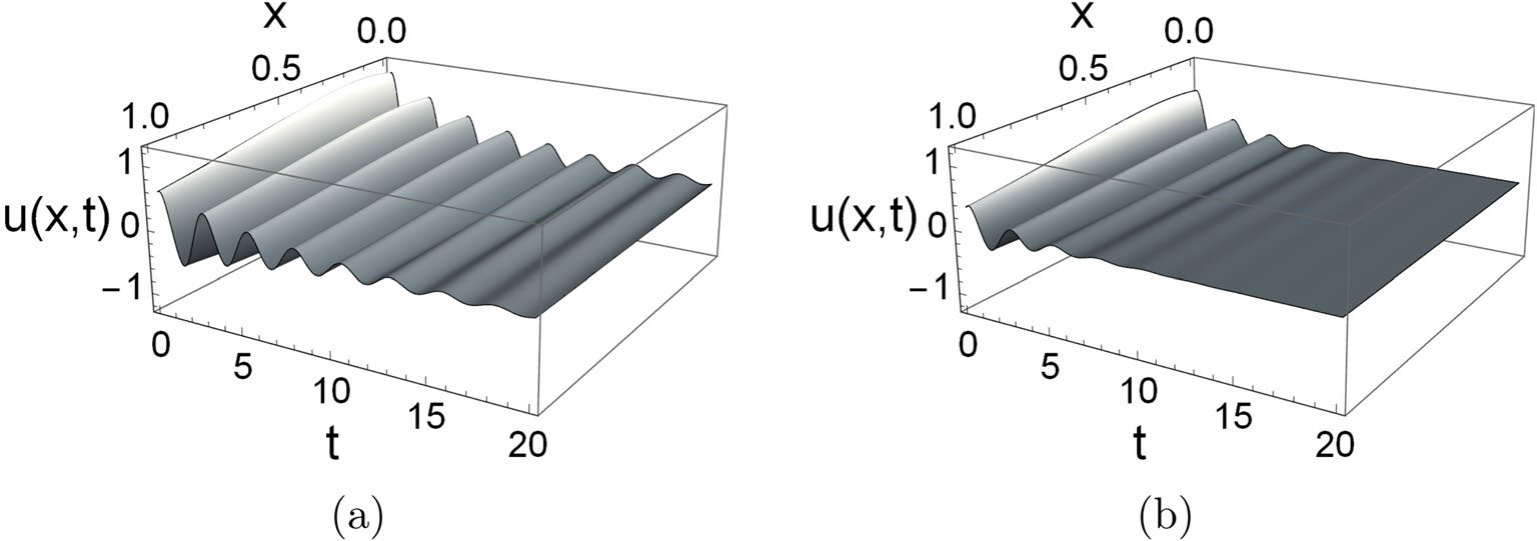
Solution *u*(*x*, *t*) corresponding to the first mode shape for exponentially changing rod stiffness with damping values (**a**) $d_{0}=0.25$ and $d_{1}=0$, and (**b**) $d_{0}=0$ and $d_{1}=0.25$.

In this case, $T_1(0)$ can be chosen arbitrarily since it is only a constant multiplier of the solution. The solution *u*(*x*, *t*) is depicted in Fig. 7 for two damping combinations.

## Optimal damping

The damping of a particular vibrational system is considered optimal if the decay of the vibration is the fastest (oscillatory motion is assumed, the overdamped behavior is not investigated). Nakic^5^ presents different approaches on finding the optimal damping for vibrational systems. One of these approaches relies on the spectral abscissa criterion where the optimal damping is achieved when the real part of the rightmost eigenvalue is minimal, i.e., where $\text {max} \{\alpha _{j}\}$ is minimal (for a wealth of applications see^56^).

This definition is straightforward when the vibrational system has only one damper. When one of the damping coefficients is zero in our rod model, the optimal damping based on the spectral abscissa criterion is the following:

- When $d_{0}=0$, $d_{1}=d_{1,opt}$ is the optimal damping.
- When $d_{1}=0$, $d_{0}=d_{0,opt}$ is the optimal damping.

For the constant stiffness rod ($k(x)\equiv 1$) $d_{0,opt} =d_{1,opt}$. In general, for any functionally graded rod $d_{0,opt}\ne d_{1,opt}$ is expected. When both dampers are included in the model, the optimal damping is provided by the damping parameters $d_{0}=\tilde{d} _{0,opt}$, $d_{1}=\tilde{d}_{1,opt}$ for which $\text {max}\{\alpha _{j}\}$ is minimal. In general, these damping parameters are not expected to be equal with the aforementioned $d_{0,opt},\,d_{1,opt}$, i.e., $\tilde{d}_{0,opt}\ne d_{0,opt}$, and $\tilde{d}_{1,opt}\ne d_{1,opt}$.

**Figure 8:**
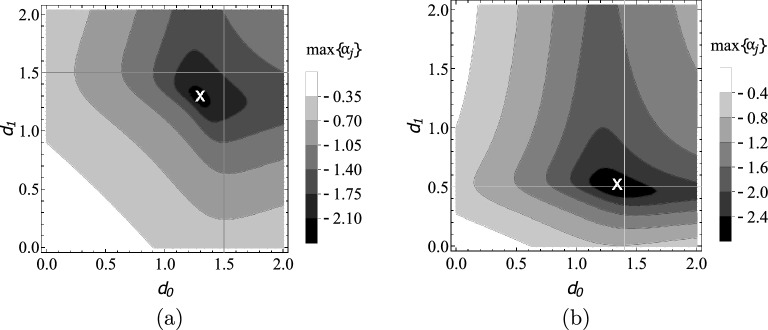
Contours of max$\{\alpha _{j}\}$ as function of the damping parameters $d_{0},\,d_{1}$ for stiffness distributions (**a**) $k(x)=1$, and (**b**) $k(x)=e^{2x}$.

In Fig. 8 the contours of $\text {max} \{\alpha _{j}\}$ are shown for a constant stiffness rod and a functionally graded rod with exponential stiffness distribution as function of $d_{0}$ and $d_{1}$. The thin vertical and horizontal lines mark $d_{0,opt}$ and $d_{1,opt}$, respectively, while the white cross marks the point $[\tilde{d}_{0,opt},\tilde{d}_{1,opt}]$.

## Energy and its distribution in the rod

In the previous “The functionally graded elastic rod model”–“The constant stiffness rod and graded rods” section we showed how to compute the eigenvalues and mode shapes of the functionally graded rod for non-constant stiffness distributions and damping parameters. Now we can compute the energy measures of the system based on those results.

The total dimensionless energy is the sum of the energy stored in the rod and the energy stored in the springs. The energies stored in the two springs are

25 $$\begin{aligned} E_{\textrm{spring,left}}(t)=\frac{1}{2}k_{0}u^{2}(0,t),\quad E_{\textrm{spring,right}}(t)=\frac{1}{2}k_{1}u^{2}(1,t). \end{aligned}$$

The energy density distribution of the rod is given by the sum of the kinetic and potential energy densities of a cross-section at location *x* at time *t*, i.e.,

26 $$\begin{aligned} \begin{aligned} e(x,t)&=e_{\textrm{kin}}(x,t)+e_{\textrm{pot}}(x,t)=\frac{1}{2}\left( \frac{\partial u(x,t)}{\partial t}\right) ^{2}+\frac{1}{2}k(x)\left( \frac{\partial u(x,t)}{\partial x}\right) ^{2},\\ e_{\textrm{kin}}(x,t)&=\frac{1}{2}\left( \frac{\partial u(x,t)}{\partial t}\right) ^{2},\quad e_{\textrm{pot}}(x,t)=\frac{1}{2}k(x)\left( \frac{\partial u(x,t)}{\partial x}\right) ^{2}. \end{aligned} \end{aligned}$$

Equation (26) captures the temporal distribution of energy along the length of the rod. The total energy $E_{\textrm{rod}}(t)$ stored in the rod at time *t* equals to the spatial integral of *e*(*x*, *t*) over the length of the rod, while the total energy $E_{\textrm{total}}(t)$ of the entire system (3) at time *t* is the sum of the energy of the springs and the energy of the rod, i.e.,

27 $$\begin{aligned} \begin{aligned} E_{\textrm{rod}}(t)&=\int \limits _{0}^{1}e(x,t){\textrm{d}}x,\\ E_{\textrm{total}}(t)&=E_{\textrm{rod}}(t)+E_{\textrm{spring,left} }(t)+E_{\textrm{spring,right}}(t). \end{aligned} \end{aligned}$$

Now we can define the scaled energy $\hat{E}_{rod}(t)$ of the rod and the scaled energy density distribution $\hat{e}(x,t)$ of the rod as

28 $$\begin{aligned} \hat{E}_{rod}(t)=\frac{1}{l}\int \limits _{0}^{1}\hat{e}(x,t){\textrm{d}} x=\frac{E_{\textrm{rod}}(t)}{E_{\textrm{total}}(t)},\quad \hat{e} (x,t)=\frac{l\cdot e(x,t)}{E_{\textrm{total}}(t)}, \end{aligned}$$

where $l=1$ is the dimensionless length of the rod. Thus, the sum of the scaled energies of the rod and the springs are equal to 1, i.e.,

29 $$\begin{aligned} \frac{1}{l}\int \limits _{0}^{1}\hat{e}(x,t){\textrm{d}}x+\frac{E_{\textrm{spring,left}}(t)}{E_{\textrm{total}}(t)}+\frac{E_{\textrm{spring,right}}(t)}{E_{\textrm{total}}(t)}=1. \end{aligned}$$

## Energy density distribution of the damped system with varying stiffness

The eigenvalues $\lambda _{\pm 1}=\alpha _{1}\pm i\omega _{1}$ with the least negative real part are associated with the slowest decaying motion. After sufficiently long time, the mode shapes $W(x,\lambda _{1}),\, Q(x,\lambda _{1})$ associated with the rightmost eigenvalue pair $\lambda _{1},\,\bar{\lambda }_{1}$ will be the dominant one and thus properly characterizes the vibration in the asymptotic limit $t\rightarrow \infty$.

We define the mean of the scaled energy of the rod ($\bar{E}_{\textrm{rod}}(\omega _{1})$) as as well as the mean of the scaled energy density distribution of the rod ($\bar{e}(x,\omega _{1})$) in the asymptotic limit averaged over the oscillation period $\omega _{1}/\pi$:

30 $$\begin{aligned} {\bar{E}}_{\textrm{rod}}(\omega _{1})=\lim \limits _{\tau \rightarrow \infty } \frac{\omega _{1}}{\pi }\int \limits _{\tau }^{\tau +\frac{\pi }{\omega _{1}}}\hat{E} _{rod}(t)\,{\textrm{d}}t,\quad \bar{e}(x,\omega _{1})=\lim \limits _{\tau \rightarrow \infty }\frac{\omega _{1}}{\pi }\int \limits _{\tau }^{\tau +\frac{\pi }{\omega _{1}}}\hat{e}(x,t)\,{\textrm{d}}t. \end{aligned}$$

The energy measure $\bar{e}(x,\omega _{1})$ characterizes the asymptotic behavior of the system, it shows us how the energy tends to distribute along the rod during free vibration.

We can compute the mean scaled energy density distribution $\bar{e}(x,\omega _{1})$ and the mean scaled energy of the rod $\bar{E}_{\textrm{rod}}(\omega _{1})$ as defined by Eq. (30) of rods having different stiffness distributions for different combinations of the damping values $d_{0},\,d_{1}$. To compute $\bar{e}(x,\omega _{1})$ and $\bar{E}_{rod}(\omega _{1})$, it is convenient to compute *u*(*x*, *t*) by choosing the initial conditions as

31 $$\begin{aligned} f(0)=W(0,\lambda _{1}),\quad g(0)=\alpha _{1}W(0,\lambda _{1})+\omega _{1}Q(0,\lambda _{1}). \end{aligned}$$

For this natural choice of initial conditions we can have $T_{1}(0)=1$ and it is ensured that the boundary conditions satisfy (14). With initial conditions (31), the mean scaled energy $\bar{E}_{\textrm{rod}} (\omega _{1})$ of the rod and the mean scaled energy density distribution $\bar{e}(x,\omega _{1})$ can simply be calculated as

32 $$\begin{aligned} \bar{E}_{\textrm{rod}}(\omega _{1})=\frac{\omega _{1}}{\pi }\int \limits _{0}^{\frac{\pi }{\omega _{1}}}\hat{E}_{rod}(t)\,{\textrm{d}}t,\quad \bar{e}(x,\omega _{1} )=\frac{\omega _{1}}{\pi }\int \limits _{0}^{\frac{\pi }{\omega _{1}}}\hat{e} (x,t)\,{\textrm{d}}t. \end{aligned}$$

**Figure 9:**
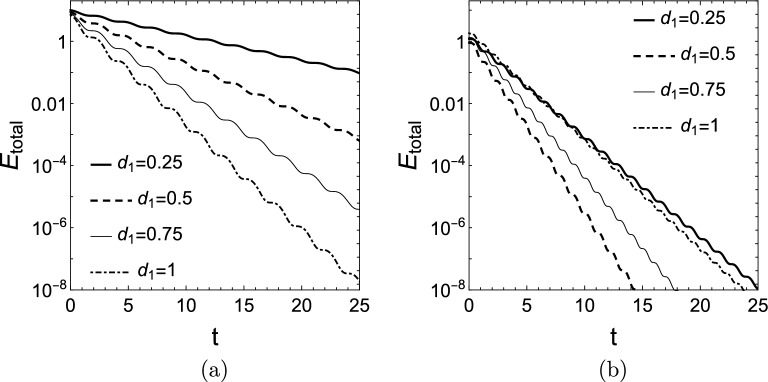
Total energy $E_{total}(t)$ of the system (3) for (**a**) constant stiffness elastic rod, $k(x)=1,d_{0} =0$, and (**b**) for a functionally graded elastic rod, $k(x)=e^{2x},d_{0}=0$.

Figure 9 shows how the total energy $E_{\textrm{total}}(t)$ of the system decays for homogeneous and a functionally graded elastic rod with different damping values. In the cases shown in Fig. 9a the dissipation rate of the total energy is gradually increasing as the damping increases. In Fig. 9b we see that the value of $d_{1,opt}$ must be in the investigated range as the decay rate starts to decrease past $d_{1}=0.5$. Indeed, either from Figs. 5b or 8b we see that for the exponential stiffness distribution $k(x)=e^{2x}$ with $d_{0}=0$ the value of the optimal damping is about $d_{1,opt}\approx 0.5$.

**Figure 10:**
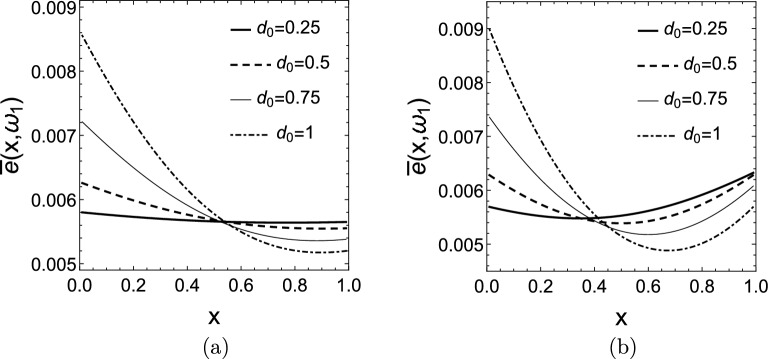
Mean scaled energy density distributions $\bar{e}(x,\omega _{1})$ for $k(x)=1$ and $d_{0}=0.25-1$, while (**a**) $d_{1}=0$, (**b**) $d_{1}=0.5$.

We now show how the mean scaled energy density distribution $\bar{e}(x,\omega _{1})$ changes as function of the damping values $d_{0},d_{1}$ for constant and varying stiffness distributions. Figure 10 shows $\bar{e}(x,\omega _{1})$ for $k(x)=1$, the parameter of the curves is $d_{0}$ which is the damping coefficient of the damper attached to the left end of the rod. In the cases depicted in Fig. 10a and b the right damping was set to $d_{1}=0$, $d_{1}=0.5$, respectively. The graphs show that increasing the damping in one end increases the energy fraction stored in that half of the rod. For $d_{0}=d_{1}$ the distribution of $\bar{e}(x,\omega _{1})$ is symmetric as expected.

**Figure 11:**
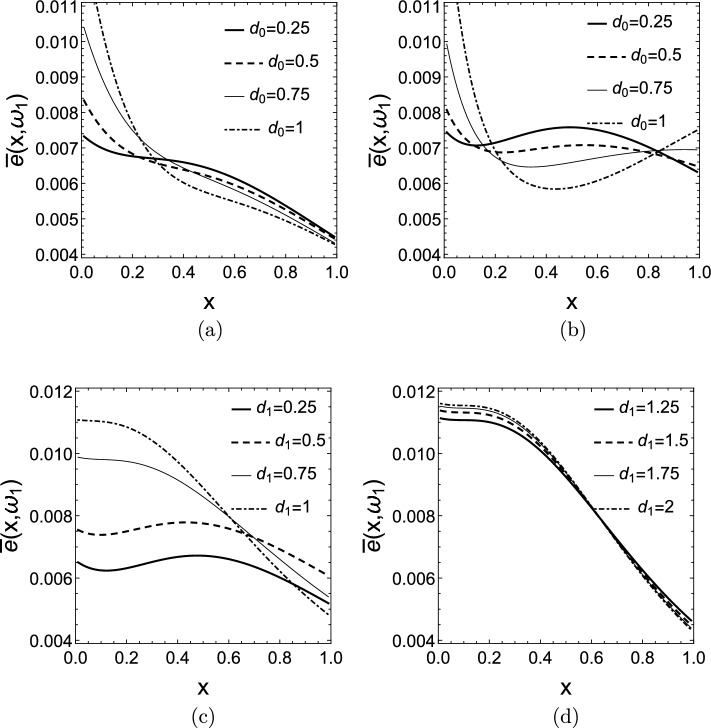
Mean scaled energy density distributions $\bar{e}(x,\omega _{1})$ for $k(x)=e^{2x}$ and (**a**) $d_{0}=0.25-1$, $d_{1}=0$, (**b**) $d_{0}=0.25-1$, $d_{1}=0.5$, (**c**) $d_{0}=0$, $d_{1}=0.25-1$, (**d**) $d_{0}=0$, $d_{1}=1.25-2$.

Figure 11a and b show $\bar{e}(x,\omega _{1})$ for $k(x)=e^{2x}$, the parameter of the curves is $d_{0}$, the other damping is set to $d_{1}=0$ and $d_{1}=0.5$, respectively. Again, in most cases we see that the energy distribution tends to be higher close to the more strongly damped end of the rod. When $d_{1}=0.5$, the $d_{0}\le d_{1}$ cases show that the energy content of both half of the rod is more or less equal, but for $d_{1}>d_{0}$ the energy content of the left half of the rod -to which this stronger damper is attached- increases again.

In Fig. 11c and d $\bar{e}(x,\omega _{1})$ graphs are depicted again for $k(x)=e^{2x}$, but this time the parameter of the curves is $d_{1}$ and $d_{0}=0$ is set. As the damping $d_{1}$ increases on the stiff end, the energy distribution becomes highly skewed and most of the energy is concentrated in the softer half of the rod. Below the optimal damping $d_{1,opt}\approx 0.5$ the energy distributions are less skewed and contain an inflection point.

**Figure 12:**
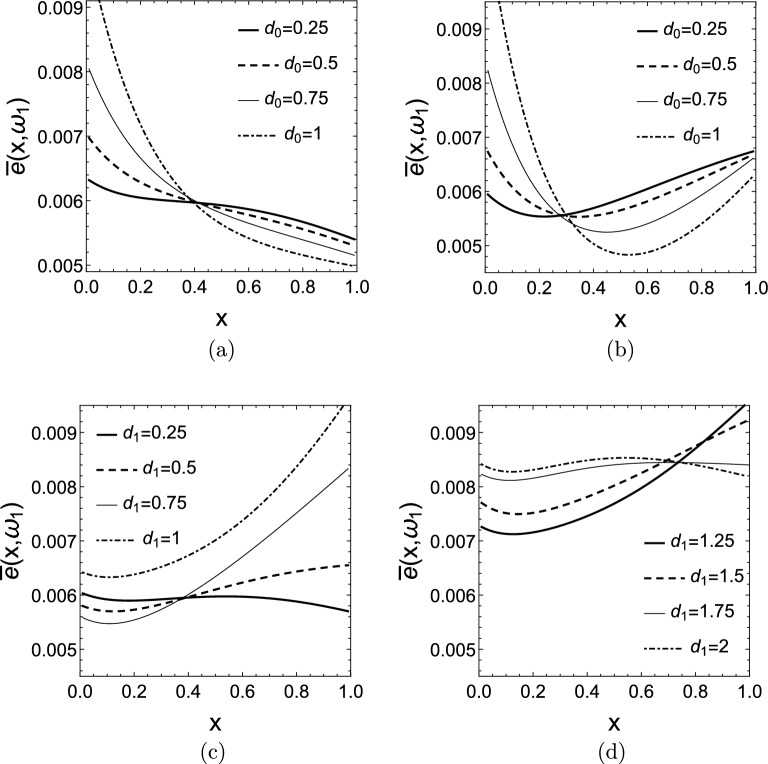
Mean scaled energy density distributions $\bar{e}(x,\omega _{1})$ for $k(x)=1+1.5x$ and (**a**) $d_{0}=0.25-1$, $d_{1}=0$, (**b**) $d_{0}=0.25-1$, $d_{1}=0.5$, (**c**) $d_{0}=0$, $d_{1}=0.25-1$, (**d**) $d_{0}=0$, $d_{1}=1.25-2$.

All of the calculations were also carried out for a milder linear stiffness distribution that has the form $k(x)=1+1.5x$ to obtain more general conclusions. Figure 12a and b show $\bar{e}(x,\omega _{1})$ for $k(x)=1+1.5x$, the parameter of the curves is $d_{0}$. The other damping is set to $d_{1}=0$ and $d_{1}=0.5$, respectively. Similarly to the constant stiffness rod, we see that the energy distribution tends to be higher close to the more strongly damped end of the rod.

In Fig. 12c and d $\bar{e}(x,\omega _{1})$ graphs are depicted for $k(x)=1+1.5x$, but this time the parameter of the curves is $d_{1}$ and $d_{0}=0$ is set. Initially, we can see that the energy distribution increases in the more strongly damped stiff part of the rod. A qualitative change is observed in the energy distribution by the value of $d_{1}=1$. The reason for this is that the damping $d_{1}=1$ already exceeds the optimal damping $d_{1,opt}$ for this case that leads to less efficient energy dissipation. A significantly smaller fraction of energy is passed to the springs connected parallel with the dampers, this is why the total mean scaled energy $\bar{E}(\omega _{1})$ of the rod is higher (the entire $\bar{e}(x,\omega _{1})$ graph is lifted compared to the $d_{1}\le d_{1,opt}$ cases). Eventually, the energy distribution tends to gravitate towards a more uniform distribution.

These results show that increasing the damping at the soft end will generally increase the energy fraction stored in the softer part of the rod. That is, the energy distribution can be manipulated along the rod by tuning the damping at the soft end. One can also see that the mean energy density distribution is qualitatively different in the case of inhomogeneous rods compared to the homogeneous rod. For a homogeneous rod we have a convex distribution in every case, whereas for the inhomogeneous cases there is an inflection point in multiple cases.

On the other hand, increasing the damping at the stiff end leads to very different energy distributions depending on the stiffness distribution of the rod. By the linear stiffness distribution this increased the energy fraction stored in the stiff part of the rod, but by the exponential stiffness distribution the effect was completely the opposite. This implies that rods with such a highly inhomogeneous stiffness distribution are very sensitive to the variation of the damping at the stiff end of the rod.

## Conclusions

The general mathematical description of the longitudinal vibration of functionally graded elastic rods with viscous and elastic boundary conditions was provided. The characteristic equation was derived for the undamped case. A homotopy-based approach to solve the equations describing the longitudinal vibration of functionally graded elastic rods was proposed. First, the solution for the undamped constant stiffness rod was calculated, then the homotopy method was used to determine the eigenvalues and mode shapes corresponding to graded rods with damping.

It was demonstrated that the method works for combined viscous and elastic boundary conditions for different types of rod stiffness distributions and a wide range of damping parameters. We carried out the computations for homogeneous rod and rods with exponentially and linearly changing stiffness. We showed the rightmost six eigenvalues and the mode shape associated with the rightmost eigenvalue for the homogeneous rod and the rod with exponentially changing stiffness.

An energy measure, the mean scaled energy density distribution was derived to compare the energy density distribution of different functionally graded elastic rods during the longitudinal vibration of the system. Besides the constant stiffness rod, two examples were provided: one with linearly changing stiffness and another with exponentially changing stiffness. It was shown that below the optimal damping the increase of the damping at one end generally makes the rod accumulate more energy close to that end. The only exception was by the most extreme case, the exponential stiffness distribution. In this case, we could not force the system to increase the relative energy content close to the stiff end.

Above the optimal damping, where the decay rate of the vibration is the highest, the energy distributions become very similar to each other, further increasing the damping does not lead to any significant changes. It was also found that there is usually an inflection point in the energy distribution of the inhomogeneous rods, while there is not any in that of the homogeneous rod that has constant stiffness. These results suggest that the energy distribution of functionally graded rods can be manipulated with the tuning of the damping parameters.

In future work, the rod model will be extended to include nonlinear boundary conditions and nonlinear attachments (e.g., nonlinear energy sinks at certain rod positions). We want to study the targeted energy transfer between the inhomogeneous rod and the nonlinear attachment to achieve the fastest decay rate in the system. We also want to investigate the effect of external forcing and how it affects the energy distribution of the system. We also intend to extend the results by investigating the behavior of the model for further combinations of gradings and damping parameters.

### Supplementary Information

Supplementary Information.

## Data Availability

The datasets generated during and/or analyzed during the current study are available from the corresponding author on reasonable request.

## List of symbols

*A*: Area of cross section [$\text {m}^2$]
$c_0,c_L$: Damping at the two ends of the rod [$\text {kg/s}$]
$d_0,d_1$: Dimensionless damping at the two ends of the rod [1]
$D_1,D_2$: Coefficients of the general solution [1]
$e(x,t),\hat{e}(x,t)$: Dimensionless energy distribution and scaled energy distribution [1]
$E,\hat{E},\bar{E}$: Dimensionless, scaled and averaged energy [1]
*f*(*x*): Nondimensional initial displacement [1]
*g*(*x*): Nondimensional initial velocity [1]
$k(\xi ),k(x)$: Function of the variation of the Young’s modulus [1]
$k_0,k_1$: Dimensionless spring stiffness at the two ends of the rod [1]
*L*: Length of the rod [$\text {m}$]
$P_c,P_g$: Characteristic polynomials of the constant stiffness and graded rods [$\text {s}$]
$Q(x,\lambda )$: Real mode shape associated with the imaginary part of $U(x,\lambda )$ [$\text {1}$]
$s_0,s_L$: Stiffness of the springs at the two ends of the rod [$\text {kg/s}^2$]
*t*: Dimensionless time [$$\text {1}$$]
$T(t,\lambda )$: Temporal part of the solution [1]
*u*(*x*, *t*): Dimensionless displacement in longitudinal direction [$\text {1}$]
$$u(x,t,\lambda )$$: Partial solution [$$\text {1}$$]
$U(x,\lambda )$: Complex mode shape [$\text {1}$]
$W(x,\lambda )$: Real mode shape associated with the real part of $U(x,\lambda )$ [$\text {1}$]
*x*: Dimensionless axial coordinate $\left[\text {1}\right]$
$\alpha , \alpha _{1}$: Real part of the eigenvalue and that of the rightmost eigenvalue [1]
$\varepsilon (\xi )$: Function of the Young’s modulus [Pa]
$\varepsilon _{0},\varepsilon _{L}$: Values of the Young’s modulus at the two ends of the rod [Pa]
$\Theta$: Timescale of the system [$\text {s}$]
$\lambda , \lambda _{1}$: Eigenvalue and rightmost eigenvalue [1]
$$\mu$$: Grading coefficient [1]
$$\xi$$: Axial coordinate [$$\text {m}$$]
$$\rho$$: Density [$$\text {kg}/\text {m}^3$$]
$$\tau$$: Time [$$\text {s}$$]
$\phi (\xi ,\tau )$: Displacement in longitudinal direction [$\text {m}$]
$\varphi (x,t,\lambda )$: Function used in Eq. (12) [$\text {1}$]
$\Psi (x,\lambda )$: Function of the general solution [1]
$\omega , \omega _{1}$: Imaginary part of the eigenvalue and that of the rightmost eigenvalue [1]
