# Supplementary material for: Vibrations and energy distribution in inhomogeneous rods with elastic and viscous boundary conditions
Source: Sci Rep. 2024 Feb 3;14:2846. doi: 10.1038/s41598-024-52860-4 (PMC10838305; doi:10.1038/s41598-024-52860-4)
Supplement: Supplementary file 1 — Supplementary Information. [file 41598_2024_52860_MOESM1_ESM.pdf]

Supplementary Information for the manuscript titled Vibrations and energy distribution in inhomogeneous rods with elastic and viscous boundary conditions

János Lelkes[1], Bendegúz Dezső Bak[1] & Tamás Kalmár-Nagy[1]

[1]Department of Fluid Mechanics, Faculty of Mechanical Engineering,

Budapest University of Technology and Economics,

Műegyetem rkp. 3., H-1111 Budapest, Hungary

November 6, 2023

Appendix - Homotopy continuation for eigenvalues

To calculate the eigenvalues λ_j corresponding to the arbitrary stiffness distribution $k(x)$ and d_0, d_1 damping values, a three-step homotopy method was applied. In the first step, the purely imaginary eigenvalues of the constant stiffness rod without damping were determined by solving Eq. (18), i.e.,

$$P_c(\lambda_j, 0, 0) = \left(\lambda_j^2 + 1\right)\sinh(\lambda_j) + 2\lambda\cosh(\lambda_j) = 0. \tag{S.1}$$

The first homotopy was applied to calculate the eigenvalues of the graded but undamped rod. For this, the homotopy was constructed as

$$\mathcal{H}_\mu(\lambda_j, q) = (1-q)P_c(\lambda_j, 0, 0) + qP_g(\lambda_j, 0, 0), \tag{S.2}$$

where q is the homotopy parameter. The basic idea is that one can now increase q from 0 by a small amount $\delta q \ll 1$ and find the roots of $\mathcal{H}_\mu(\lambda_j, \delta q) = 0$ using a nonlinear solver by using the roots of $\mathcal{H}_\mu(\lambda_j, 0) = 0$ as the initial guess. Once the roots of $\mathcal{H}_\mu(\lambda_j, \delta q) = 0$ are found within some tolerance, q can be increased again by δq and using these roots as the initial guess $\mathcal{H}_\mu(\lambda_j, 2\delta q) = 0$

can be solved. This procedure is then continued until $q = 1$ is reached, and the roots of $P_g(\lambda_j, 0, 0)$ (see Eq. (10)) are determined.

To calculate the eigenvalues of the graded rod with dampers on both ends, two more Homotopy steps were applied. The procedure is analogous to the previous one; the second and the third homotopy equations are

$$\mathcal{H}_{d_0}(\lambda_j, q) = (1-q)P_g(\lambda_j, 0, 0) + qP_g(\lambda_j, d_0, 0), \tag{S.3}$$

$$\mathcal{H}_{d_1}(\lambda_j, q) = (1-q)P_g(\lambda_j, d_0, 0) + qP_g(\lambda_j, d_0, d_1). \tag{S.4}$$

At the end of the third Homotopy procedure, the roots of $P_g(\lambda_j, d_0, d_1)$ are determined.
